# Association between sarcopenia and the risk of diabetic cardiomyopathy in patients with type 2 diabetes mellitus: a single-center retrospective cohort study

**DOI:** 10.3389/fgene.2026.1892826

**Published:** 2026-08-25

**Authors:** Xia Liu, Lingjie Xu, Li Zhang, Hailin Yang, Qian Xiao

**Affiliations:** 1 Department of Geriatrics, Laboratory of Research and Translation for Geriatric Diseases, The First Affiliated Hospital of Chongqing Medical University, Chongqing, China; 2 Shapingba Hospital Affiliated to Chongqing University (Shapingba District People’s Hospital of Chongqing), Chongqing, China; 3 Department of General Practice, The First Affiliated Hospital of Chongqing Medical University, Chongqing, China

**Keywords:** AWGS 2019, AWGS 2025, diabetic cardiomyopathy, ferritin, inflammatory biomarkers, muscle-heart axis, retrospective cohort study, sarcopenia

## Abstract

**Background:**

Sarcopenia is an emerging complication of type 2 diabetes mellitus (T2DM) characterized by progressive loss of skeletal muscle mass and function. Diabetic cardiomyopathy (DCM) is a distinct cardiac disease in T2DM patients independent of coronary artery disease and hypertension. Whether sarcopenia independently predicts DCM risk in T2DM patients, and whether shared molecular pathways including systemic inflammation and metabolic dysregulation underlie this association, remains unclear.

**Methods:**

This retrospective cohort study enrolled 400 T2DM patients admitted to the First Affiliated Hospital of Chongqing Medical University between February 2022 and February 2026. Sarcopenia was diagnosed using AWGS 2019 criteria as the primary framework, with concurrent evaluation against the updated AWGS 2025 criteria. DCM was diagnosed by Doppler echocardiography in T2DM patients with myocardial dysfunction after excluding other cardiac etiologies, per clinically established criteria (2024 ESC DCM position statement); this echocardiographic approach, while standard practice, does not carry the tissue-characterization precision of cardiac MRI. Multivariate logistic regression (three sequentially adjusted models), propensity score matching, and prespecified subgroup analyses were performed.

**Results:**

Sarcopenia was identified in 137/400 patients (34.3%) and DCM in 113/400 (28.3%). DCM prevalence was significantly higher in sarcopenic versus non-sarcopenic patients (44.5% vs. 19.8%, p < 0.001). After full adjustment, sarcopenia remained independently associated with DCM risk (adjusted OR = 2.70, 95% CI: 1.72–4.24, p < 0.001; AUC-ROC = 0.812). The association was consistent across all subgroups (all p-interaction > 0.05). Sarcopenic patients exhibited elevated hs-CRP, NT-proBNP, ferritin, and uric acid, with lower vitamin D levels.

**Conclusion:**

Sarcopenia is independently associated with significantly elevated DCM risk in T2DM patients; the cross-sectional design precludes definitive causal inference and directionality requires prospective longitudinal confirmation. Shared mechanisms—including systemic inflammation, elevated ferritin and uric acid, and potentially common genetic regulatory pathways (hypothesized on the basis of existing literature)—may underlie the muscle–heart dysfunction axis. Routine sarcopenia screening using AWGS 2019 criteria—consistent with the updated AWGS 2025 framework—may facilitate early identification of T2DM patients at elevated DCM risk.

## 1 Introduction

Type 2 diabetes mellitus (T2DM) represents a global pandemic affecting over 537 million adults, with projections exceeding 783 million by 2045 (ElSayed et al., 2023; Magliano and Boyko, 2022). Among its numerous complications, diabetic cardiomyopathy (DCM) has emerged as a clinically significant yet underrecognized entity—defined as systolic and/or diastolic myocardial dysfunction in patients with T2DM in the absence of coronary artery disease, hypertension, valvular disease, or other known cardiac etiologies (Jia and Sowers, 2020; Seferovic and Paulus, 2015). DCM substantially increases the risk of heart failure and cardiovascular mortality, yet lacks specific early biomarkers for risk stratification (Seferovic and Paulus, 2015; Seferović et al., 2024).

Sarcopenia, characterized by progressive loss of skeletal muscle mass accompanied by reduced muscle strength and/or physical performance, has been increasingly recognized as a clinically important complication of T2DM (Chen et al., 2023). The prevalence of sarcopenia in T2DM populations ranges from 10% to 35% depending on the diagnostic criteria and study population (Chen et al., 2020; Alfaro-Alvarado et al., 2023; Shatila et al., 2024; Lu et al., 2023; Tan et al., 2022). Insulin resistance promotes muscle protein catabolism and impairs myogenesis, while reduced muscle mass exacerbates glycemic dysregulation by diminishing the primary tissue for insulin-mediated glucose disposal (Chen et al., 2023; Purnamasari et al., 2022). This bidirectional relationship creates a vicious cycle that accelerates both metabolic and functional decline.

The biological plausibility of a sarcopenia-DCM link in T2DM is supported by shared molecular pathways. Skeletal muscle functions as an endocrine organ secreting myokines that exert significant paracrine and endocrine effects on cardiomyocyte metabolism and cardiac remodeling (Severinsen and Pedersen, 2020). Chronic low-grade inflammation, a hallmark of both sarcopenia and DCM, simultaneously drives skeletal muscle protein catabolism and cardiac fibroblast activation, and may be reflected by elevated systemic markers such as hs-CRP, ferritin, and uric acid (Tan et al., 2020; Mittal et al., 2021). Furthermore, the relationship between metabolic dysregulation and cardiac dysfunction in T2DM may be mediated by common inflammatory and oxidative pathways, suggesting overlapping genetic and epigenetic regulatory mechanisms (Tan et al., 2020; Mittal et al., 2021).

Despite growing evidence linking sarcopenia with increased cardiovascular risk in T2DM (Boonpor et al., 2024; Wei et al., 2024; Park et al., 2021), studies specifically examining the sarcopenia-DCM association remain limited. The present retrospective cohort study therefore aimed to: (i) determine sarcopenia and DCM prevalence in a well-characterized T2DM cohort admitted between February 2022 and February 2026; (ii) evaluate the independent association between sarcopenia and DCM risk after comprehensive covariate adjustment; (iii) characterize differences in inflammatory, myokine, and cardiac biomarkers; and (iv) assess subgroup consistency.

## 2 Materials and methods

### 2.1 Study design and setting

This single-center retrospective cohort study was conducted at the First Affiliated Hospital of Chongqing Medical University, China. Medical records of T2DM patients admitted to the Departments of Geriatrics, Endocrinology, and General Practice between February 2022 and February 2026 were systematically reviewed. The study was approved by the Institutional Review Board (Approval No. K2023-085) and conducted in accordance with the Declaration of Helsinki. Individual informed consent was waived given the retrospective design.

### 2.2 Inclusion and exclusion criteria

**FIGURE 1:**
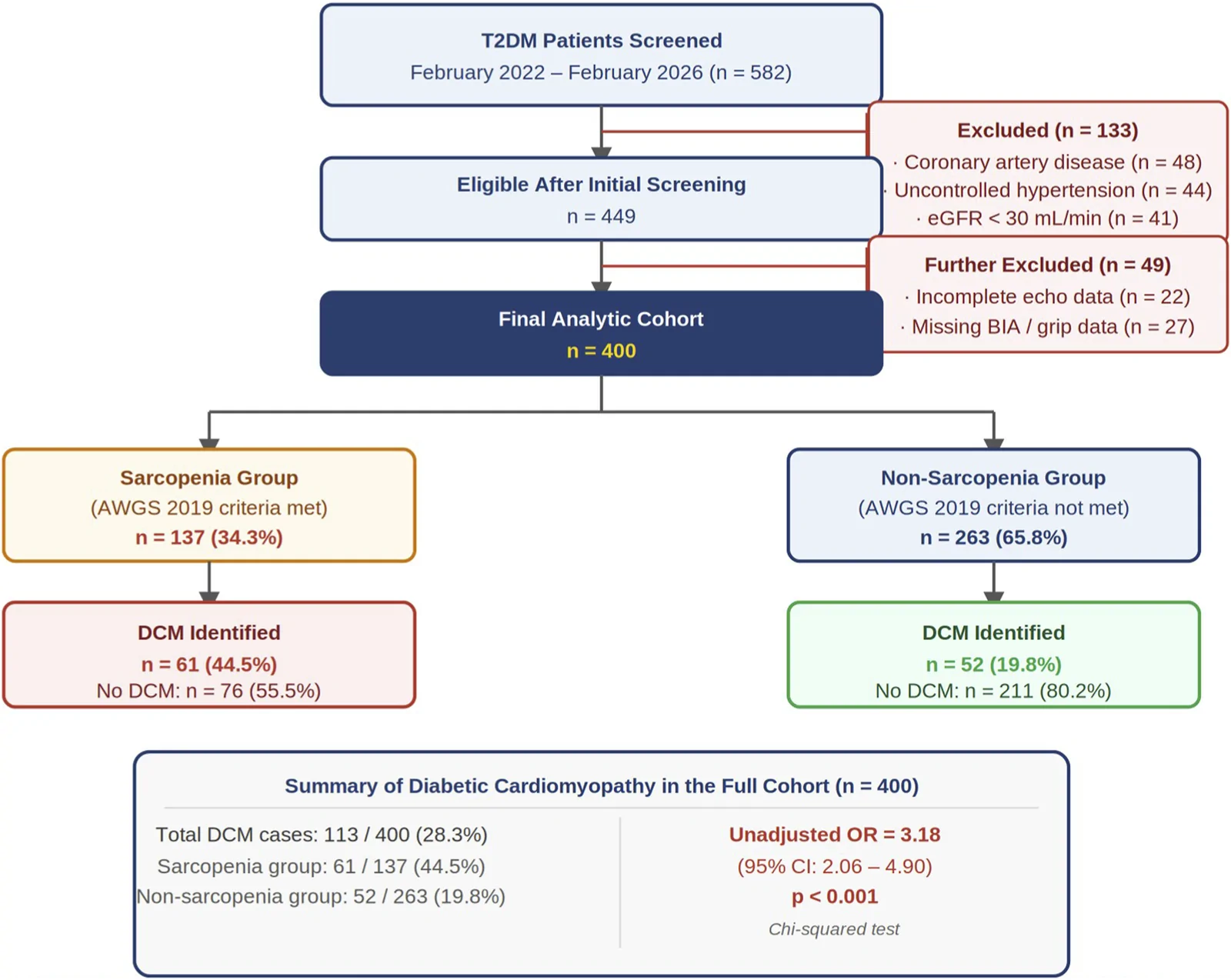
Patient selection flowchart. Of 582 T2DM patients screened between February 2022 and February 2026, 400 met all inclusion criteria and were included in the final analytic cohort. T2DM, type 2 diabetes mellitus; eGFR, estimated glomerular filtration rate; BIA, bioelectrical impedance analysis; AWGS, Asian Working Group for Sarcopenia (2019 criteria); DCM, diabetic cardiomyopathy; OR, odds ratio; CI, confidence interval.

Inclusion criteria: (1) age ≥ 18 years; (2) confirmed T2DM by 2023 ADA standards of care (ElSayed et al., 2023); (3) complete echocardiographic evaluation; (4) available BIA and handgrip strength measurements. Exclusion criteria: (1) type 1 or other specific diabetes types; (2) significant coronary artery disease (≥50% stenosis or confirmed myocardial infarction); (3) uncontrolled hypertension (defined as systolic blood pressure ≥180 mmHg and/or diastolic blood pressure ≥110 mmHg [Grade 3 hypertension] on repeated measurement at admission, consistent with the 2023 ESH/ESC Hypertension Guidelines; patients with controlled or treated hypertension [SBP < 180 mmHg] were retained and hypertension status was included as a covariate in the fully adjusted Model 3); (4) moderate-to-severe valvular heart disease; (5) primary cardiomyopathy; (6) eGFR < 30 mL/min/1.73 m^2^; (7) active malignancy; (8) severe liver disease; (9) inflammatory myopathy; (10) incomplete data (Figure 1).

### 2.3 Sarcopenia assessment (AWGS 2019 and 2025)

Sarcopenia was diagnosed using AWGS 2019 criteria (Chen et al., 2020) as the primary diagnostic framework. Muscle mass was assessed by BIA (InBody 770; InBody Co., Seoul, Korea); low muscle mass was defined as SMI < 7.0 kg/m^2^ (men) or < 5.7 kg/m^2^ (women). Muscle strength was assessed by dominant-hand handgrip with a digital dynamometer (Jamar Plus+); low strength: < 28 kg (men) or < 18 kg (women). Physical performance was evaluated by 6-m gait speed (<1.0 m/s), SPPB score (≤9), or 5-time chair stand test (≥12 s). Possible sarcopenia was defined by low strength or performance alone; confirmed sarcopenia required low muscle mass plus low strength or performance; severe sarcopenia required all three criteria (Chen et al., 2020). All cases were additionally cross-classified using the updated AWGS 2025 criteria (Chen et al., 2025), which refine diagnostic thresholds and incorporate calf circumference as an alternative screening measure; concordance between 2019 and 2025 classifications was evaluated as a secondary analysis.

### 2.4 Diagnosis of diabetic cardiomyopathy

DCM was diagnosed by Doppler echocardiography performed by experienced cardiologists blinded to sarcopenia status. Per the 2024 ESC DCM position statement, DCM was defined as myocardial systolic and/or diastolic dysfunction in T2DM patients after excluding coronary artery disease, hypertension, valvular disease, and other etiologies (Seferović et al., 2024). Diastolic dysfunction was graded using ASE/EACVI 2016 guidelines (E/A ratio, E/e' ratio, LAVI, peak TR velocity). LVEF was measured by Simpson biplane method; GLS by 2D speckle-tracking echocardiography.

### 2.5 Data collection and statistical analysis

Clinical, laboratory (HbA1c, lipids, renal function, UACR), and biomarker data (hs-CRP, ferritin, uric acid, tumor markers [CEA, AFP, CA125], NT-proBNP, hs-Troponin I, 25-OH vitamin D) were extracted from the electronic medical record system. Logistic regression was performed using three models: Model 1 (unadjusted); Model 2 (+age, sex); Model 3 (+BMI, diabetes duration, HbA1c, eGFR, UACR, SBP, insulin use, hypertension). Calibration was assessed by the Hosmer-Lemeshow test; discrimination by AUC-ROC. Propensity score matching (1:2, nearest-neighbor, caliper = 0.02) was performed as sensitivity analysis; covariate balance was assessed by standardized mean differences (SMDs) before and after matching for all six matched variables (age, sex, HbA1c, diabetes duration, eGFR, and BMI), with adequate balance defined as SMD < 0.10 (Supplementary Table S1 and Figure S1 [Love plot]). Prespecified subgroup analyses were conducted for age, sex, HbA1c, diabetes duration, and eGFR. Missing data were assessed by comparing baseline characteristics between the 49 excluded (incomplete data) and 400 included patients using Student’s t-test, chi-squared test, or Mann–Whitney U test as appropriate; missingness was considered likely at random if no statistically significant differences were found. All tests were two-sided; p < 0.05 was considered significant. Analyses used SPSS v26.0 and R v4.3.0.

## 3 Results

### 3.1 Study population

Of 582 screened patients, 182 were excluded (133 at initial screening: coronary artery disease n = 48, uncontrolled hypertension n = 44, eGFR < 30 mL/min/1.73 m^2^ n = 41; 49 for incomplete data: missing echo n = 22, missing BIA/grip n = 27), yielding a final cohort of 400 patients (Figure 1). Comparison of baseline characteristics between the 49 excluded patients and the 400 included patients revealed no statistically significant differences in age, sex, HbA1c, or diabetes duration (all p > 0.05), suggesting that missingness was likely at random and that complete-case analysis is unlikely to have introduced substantial selection bias. Mean age was 64.6 ± 10.4 years; 55.0% were male; mean diabetes duration was 9.6 ± 5.9 years (Table 1). Sarcopenic patients were significantly older, had longer diabetes duration, higher HbA1c, lower BMI, and lower eGFR. AWGS 2019 sarcopenia components are detailed in Table 2. Cross-classification using the updated AWGS 2025 criteria (Chen et al., 2025) yielded high concordance with 2019-based diagnoses (κ = 0.89), supporting the robustness of the primary classification.

**TABLE 1:** Baseline characteristics of the study population stratified by sarcopenia status.

Variable	All patients (n = 400)	Sarcopenia (n = 137)	Non-sarcopenia (n = 263)	p value
Age (years), mean ± SD	64.6 ± 10.4	69.1 ± 9.8	62.2 ± 9.9	<0.001
Age ≥ 65 years, n (%)	196 (49.0)	88 (64.2)	108 (41.1)	<0.001
Male sex, n (%)	220 (55.0)	66 (48.2)	154 (58.6)	0.046
BMI (kg/m^2^), mean ± SD	24.7 ± 3.6	23.0 ± 3.3	25.6 ± 3.5	<0.001
Diabetes duration (years), mean ± SD	9.6 ± 5.9	11.4 ± 6.2	8.6 ± 5.5	<0.001
HbA1c (%), mean ± SD	8.0 ± 1.4	8.4 ± 1.5	7.8 ± 1.3	<0.001
Fasting plasma glucose (mmol/L)	8.5 ± 2.7	9.1 ± 2.9	8.2 ± 2.5	0.010
SBP (mmHg), mean ± SD	132.8 ± 17.1	135.4 ± 17.6	131.4 ± 16.7	0.034
DBP (mmHg), mean ± SD	78.4 ± 10.4	77.2 ± 10.9	79.1 ± 10.0	0.098
Total cholesterol (mmol/L)	4.80 ± 1.07	4.68 ± 1.01	4.87 ± 1.10	0.108
LDL-cholesterol (mmol/L)	2.70 ± 0.91	2.62 ± 0.87	2.74 ± 0.93	0.226
HDL-cholesterol (mmol/L)	1.11 ± 0.27	1.08 ± 0.25	1.13 ± 0.28	0.094
Triglycerides (mmol/L)	2.16 ± 1.24	2.30 ± 1.33	2.08 ± 1.17	0.082
Creatinine (μmol/L)	83.0 ± 22.8	87.4 ± 24.3	80.6 ± 21.6	0.005
eGFR (mL/min/1.73 m^2^)	72.4 ± 18.6	67.8 ± 19.4	74.9 ± 17.8	0.002
UACR (mg/g), median (IQR)	29.2 (14.6–74.8)	35.6 (19.0–90.4)	25.8 (12.8–64.2)	0.022
Insulin use, n (%)	147 (36.8)	61 (44.5)	86 (32.7)	0.018
Metformin use, n (%)	282 (70.5)	89 (65.0)	193 (73.4)	0.076
SGLT2 inhibitor use, n (%)	92 (23.0)	26 (19.0)	66 (25.1)	0.146
Statin use, n (%)	204 (51.0)	70 (51.1)	134 (51.0)	0.986
Hypertension, n (%)	184 (46.0)	74 (54.0)	110 (41.8)	0.017
Dyslipidaemia, n (%)	228 (57.0)	78 (56.9)	150 (57.0)	0.987
Current/past smoking, n (%)	80 (20.0)	30 (21.9)	50 (19.0)	0.482

Data are mean ± SD, median (IQR), or n (%). Comparisons by Student’s t-test, Mann–Whitney U test, or chi-squared test as appropriate. BMI, body mass index; SBP, systolic blood pressure; DBP, diastolic blood pressure; eGFR, estimated glomerular filtration rate; UACR, urine albumin-to-creatinine ratio; SGLT2i, sodium–glucose cotransporter-2 inhibitor.

**TABLE 2:** AWGS 2019 sarcopenia diagnostic criteria and component distribution (with AWGS 2025 cross-classification).

Diagnostic component	Cut-off	Sarcopenia group (n = 137)	Non-sarcopenia (n = 263)	p value
Handgrip strength (kg), mean ± SD	<28 (M)/< 18 (F)	21.2 ± 5.1	31.6 ± 7.0	<0.001
Low handgrip strength, n (%)	—	137 (100.0)	0 (0.0)	<0.001
Usual gait speed (m/s), mean ± SD	<1.0	0.71 ± 0.18	1.07 ± 0.19	<0.001
Low gait speed, n (%)	—	110 (80.3)	22 (8.4)	<0.001
SPPB score, median (IQR)	≤9	8 (6–9)	11 (10–12)	<0.001
Low SPPB, n (%)	—	104 (75.9)	20 (7.6)	<0.001
5-time chair stand (s), mean ± SD	≥12	15.4 ± 3.8	10.2 ± 2.4	<0.001
SMI by BIA (kg/m^2^), mean ± SD	<7.0 (M)/< 5.7 (F)	5.88 ± 0.82	7.61 ± 1.00	<0.001
Low muscle mass, n (%)	—	137 (100.0)	0 (0.0)	<0.001
Calf circumference (cm), mean ± SD	<34 (M)/< 33 (F)	30.6 ± 2.5	34.1 ± 2.7	<0.001
Sarcopenia severity				
Possible sarcopenia, n (%)	—	32 (23.4)	—	—
Confirmed sarcopenia, n (%)	—	105 (76.6)	—	—
Severe sarcopenia, n (%)	—	50 (36.5)	—	—

M, male; F, female; SPPB, short physical performance battery; SMI, skeletal muscle index; BIA, bioelectrical impedance analysis.

### 3.2 Echocardiographic parameters and DCM prevalence

Sarcopenic patients demonstrated significantly worse diastolic indices: lower E/A ratio (0.73 ± 0.20 vs. 0.86 ± 0.22, p < 0.001), higher E/e' ratio (12.2 ± 3.7 vs. 9.6 ± 2.8, p < 0.001), larger LAVI (32.0 ± 9.5 vs. 26.8 ± 7.9 mL/m^2^, p < 0.001), and worse GLS (−16.7% vs. −19.3%, p < 0.001). LVEF did not differ significantly (61.6% vs. 62.6%, p = 0.254; Table 3). DCM was present in 113 patients (28.3%) overall, with markedly higher prevalence in the sarcopenia group (44.5% vs. 19.8%, p < 0.001). DCM prevalence stratified by baseline characteristics is shown in Table 4.

**TABLE 3:** Echocardiographic parameters and DCM prevalence by sarcopenia status.

Parameter	All (n = 400)	Sarcopenia (n = 137)	Non-sarcopenia (n = 263)	p value
LVEF (%), mean ± SD	62.2 ± 7.9	61.6 ± 8.2	62.6 ± 7.7	0.254
LVEDD (mm), mean ± SD	46.6 ± 5.1	47.2 ± 5.5	46.3 ± 4.8	0.122
LV mass index (g/m^2^), mean ± SD	84.0 ± 18.4	88.2 ± 20.0	81.8 ± 17.3	0.011
E wave (cm/s), mean ± SD	68.2 ± 16.0	63.8 ± 17.2	70.6 ± 15.2	0.002
A wave (cm/s), mean ± SD	84.4 ± 18.6	88.0 ± 19.4	82.6 ± 18.0	0.024
E/A ratio, mean ± SD	0.82 ± 0.22	0.73 ± 0.20	0.86 ± 0.22	<0.001
E/e' ratio, mean ± SD	10.5 ± 3.2	12.2 ± 3.7	9.6 ± 2.8	<0.001
LAVI (mL/m^2^), mean ± SD	28.6 ± 8.7	32.0 ± 9.5	26.8 ± 7.9	<0.001
e' lateral (cm/s), mean ± SD	8.5 ± 2.4	7.3 ± 2.2	9.2 ± 2.3	<0.001
GLS (%), mean ± SD	−18.3 ± 3.2	−16.7 ± 3.4	−19.3 ± 2.9	<0.001
LA diameter (mm), mean ± SD	36.6 ± 5.3	38.0 ± 5.7	35.9 ± 5.0	0.002
IVS thickness (mm), mean ± SD	9.8 ± 1.8	10.5 ± 1.9	9.4 ± 1.7	<0.001
TAPSE (mm), mean ± SD	22.2 ± 3.6	21.6 ± 3.7	22.5 ± 3.5	0.040
DCM present, n (%)	113 (28.3)	61 (44.5)	52 (19.8)	<0.001

LVEF, left ventricular ejection fraction; LVEDD, left ventricular end-diastolic diameter; LAVI, left atrial volume index; GLS, global longitudinal strain; IVS, interventricular septum; TAPSE, tricuspid annular plane systolic excursion; DCM, diabetic cardiomyopathy.

**TABLE 4:** Prevalence of diabetic cardiomyopathy by baseline characteristics.

Characteristic	Total n	DCM n (%)	Crude OR	95% CI	p value
Age (years)					
<65	204	34 (16.7)	Reference	—	—
≥65	196	79 (40.3)	3.30	2.07–5.25	<0.001
BMI (kg/m^2^)					
<23.0	105	21 (20.0)	Reference	—	—
23.0–27.5	204	60 (29.4)	1.67	0.97–2.89	0.064
≥27.5	91	32 (35.2)	2.17	1.18–3.99	0.013
HbA1c (%)					
<7.0	100	17 (17.0)	Reference	—	—
7.0–9.0	196	52 (26.5)	1.77	0.96–3.24	0.066
>9.0	104	44 (42.3)	3.61	1.88–6.94	<0.001
Diabetes duration (years)					
<5	78	12 (15.4)	Reference	—	—
5–10	154	36 (23.4)	1.66	0.82–3.36	0.159
>10	168	65 (38.7)	3.42	1.74–6.72	<0.001
Sarcopenia					
Non-sarcopenia	263	52 (19.8)	Reference	—	—
Sarcopenia	137	61 (44.5)	3.18	2.06–4.90	<0.001

Unadjusted ORs, from univariate logistic regression; OR, odds ratio; CI, confidence interval; DCM, diabetic cardiomyopathy.

### 3.3 Univariate and multivariate logistic regression

**FIGURE 2:**
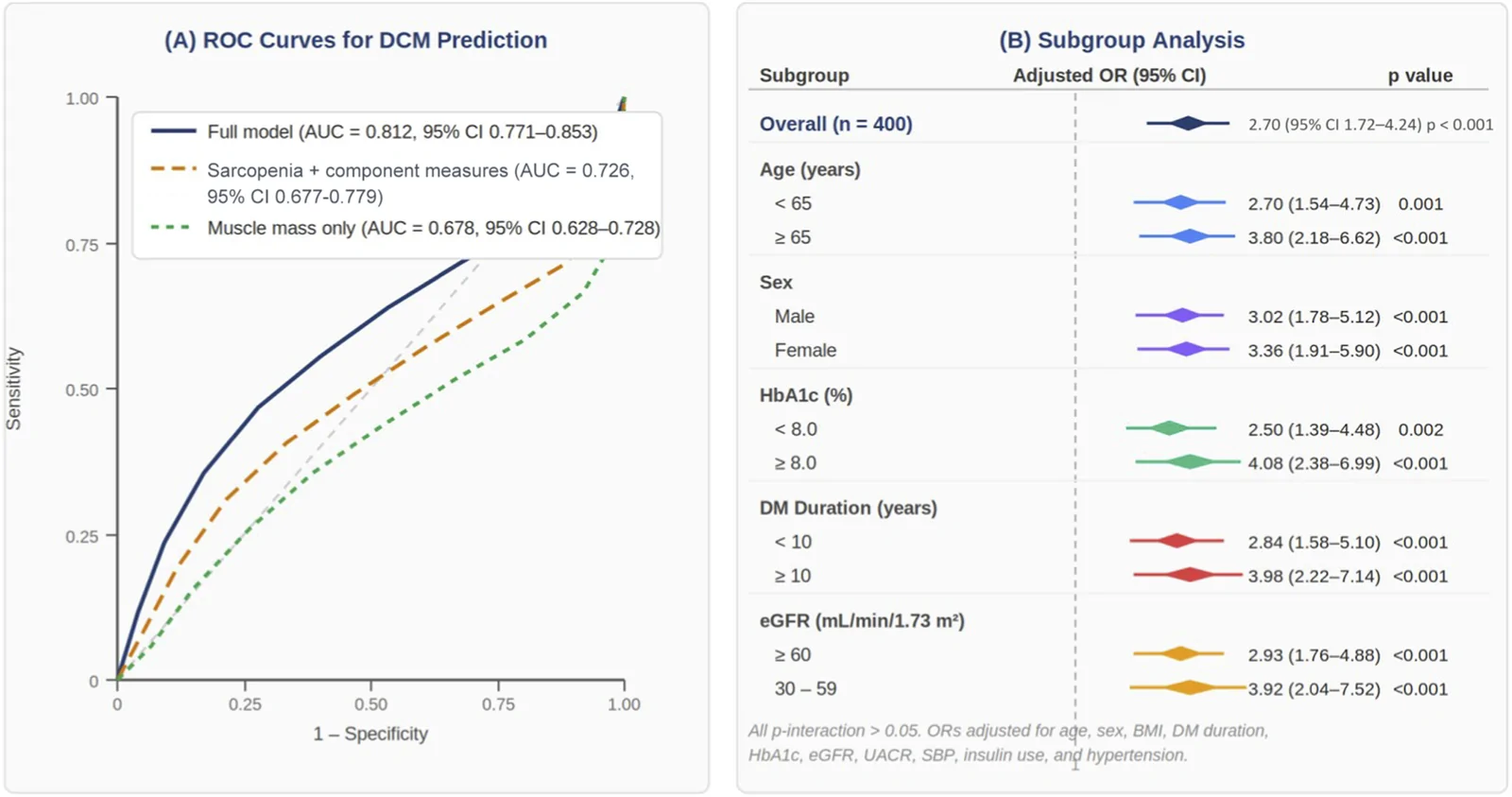
**(A)** Receiver operating characteristic (ROC) curves for three logistic regression models predicting diabetic cardiomyopathy: the full multivariate model (Model 3, navy, AUC = 0.812), a model incorporating sarcopenia (binary) plus its continuous component measures (SMI by BIA, handgrip strength, and gait speed) (orange dashed, AUC = 0.726) and muscle mass-alone model (SMI by BIA as a continuous predictor) (green dotted, AUC = 0.678). **(B)** Forest plot of prespecified subgroup analyses. All adjusted ORs are derived from Model 3 (excluding the stratification variable). Error bars represent 95% CIs. All p-interaction values > 0.05 indicate consistent association across subgroups. DM, diabetes mellitus; eGFR, estimated glomerular filtration rate; AUC, area under the curve; OR, odds ratio; CI, confidence interval.

Univariate regression identified sarcopenia, older age, longer diabetes duration, higher HbA1c, lower eGFR, elevated UACR, higher SBP, and insulin use as significant DCM predictors (Table 5). In multivariate analysis (Table 6), sarcopenia remained independently associated with DCM across all three models: unadjusted OR = 3.18 (95% CI: 2.06–4.90), age- and sex-adjusted OR = 2.82 (95% CI: 1.81–4.40), and fully adjusted OR = 2.70 (95% CI: 1.72–4.24, all p < 0.001). The fully adjusted Model 3 achieved AUC-ROC = 0.812 (95% CI: 0.771–0.853) with good calibration (Hosmer-Lemeshow p = 0.584). ROC curves are shown in Figure 2A.

**TABLE 5:** Univariate logistic regression: predictors of diabetic cardiomyopathy.

Variable	OR	95% CI	p value
Sarcopenia (yes vs. no)	3.18	2.06–4.90	<0.001
Age (per 10 years)	1.52	1.24–1.86	<0.001
Sex (female vs. male)	1.09	0.72–1.65	0.681
BMI (per kg/m^2^)	1.07	1.02–1.14	0.013
Diabetes duration (per year)	1.09	1.05–1.13	<0.001
HbA1c (per 1%)	1.34	1.19–1.51	<0.001
Fasting plasma glucose (per mmol/L)	1.11	1.03–1.19	0.005
SBP (per 10 mmHg)	1.20	1.08–1.34	0.001
Creatinine (per 10 μmol/L)	1.09	1.02–1.17	0.013
eGFR (per 10 mL/min/1.73 m^2^)	0.87	0.79–0.96	0.005
UACR (log-transformed)	1.26	1.09–1.45	0.002
LDL-cholesterol (per mmol/L)	1.04	0.88–1.24	0.640
Triglycerides (per mmol/L)	1.15	1.01–1.31	0.037
Insulin use (yes vs. no)	1.66	1.08–2.54	0.020
SGLT2 inhibitor use (yes vs. no)	0.70	0.43–1.14	0.151
Hypertension (yes vs. no)	1.58	1.06–2.37	0.026
SMI by BIA (per kg/m^2^)	0.63	0.54–0.74	<0.001
Handgrip strength (per kg)	0.94	0.92–0.97	<0.001
Gait speed (per 0.1 m/s)	0.87	0.81–0.93	<0.001

OR, odds ratio; CI, confidence interval; eGFR, estimated glomerular filtration rate; UACR, urine albumin-to-creatinine ratio; SMI, skeletal muscle index; BIA, bioelectrical impedance analysis; SGLT2i, sodium–glucose cotransporter-2 inhibitor.

**TABLE 6:** Multivariate logistic regression models for diabetic cardiomyopathy risk.

Variable	Model 1 (Unadjusted) OR (95% CI)	Model 2 (+Age, Sex) OR (95% CI)	Model 3 (Fully Adjusted) OR (95% CI)
Sarcopenia (yes vs. no)	3.18 (2.06–4.90)***	2.82 (1.81–4.40)***	2.70 (1.72–4.24)***
Age (per 10 years)	—	1.38 (1.12–1.70)**	1.30 (1.03–1.64)*
Sex (female vs. male)	—	1.02 (0.66–1.57)	0.96 (0.61–1.51)
Diabetes duration (per year)	—	—	1.07 (1.02–1.11)**
HbA1c (per 1%)	—	—	1.26 (1.11–1.43)**
eGFR (per 10 mL/min/1.73 m^2^)	—	—	0.90 (0.82–0.99)*
UACR (log-transformed)	—	—	1.20 (1.03–1.39)*
SBP (per 10 mmHg)	—	—	1.13 (1.01–1.26)*
Insulin use (yes vs. no)	—	—	1.46 (0.93–2.30)
BMI (per kg/m^2^)	—	—	1.05 (0.98–1.12)
Hypertension (yes vs. no)	—	—	1.40 (0.90–2.19)
Model statistics			
Nagelkerke R^2^	0.088	0.146	0.202
Hosmer–Lemeshow p	—	—	0.584
AUC-ROC (95% CI)	0.643 (0.594–0.691)	0.720 (0.671–0.769)	0.812 (0.771–0.853)

Model 1: sarcopenia alone (unadjusted). Model 2: additionally adjusted for age and sex. Model 3: fully adjusted. *p < 0.05; **p < 0.01; ***p < 0.001. AUC-ROC, area under the receiver operating characteristic curve.

### 3.4 Subgroup analysis

Prespecified subgroup analyses confirmed the robustness of the association (Table 7; Figure 2B). Adjusted ORs were significant in all subgroups (age, sex, HbA1c level, diabetes duration, eGFR strata). Numerically stronger associations were observed in older patients (OR = 3.80 vs. 2.70), those with HbA1c ≥ 8.0% (OR = 4.08 vs. 2.50), and diabetes duration ≥ 10 years (OR = 3.98 vs. 2.84), with no significant interaction (all p-interaction > 0.05). Propensity score-matched analysis (1:2 matching on age, sex, HbA1c, diabetes duration, eGFR, BMI) confirmed the association (matched OR = 2.44, 95% CI: 1.48–4.02, p < 0.001). All six matched covariates achieved post-matching SMDs below 0.10 (Supplementary Table S1; Supplementary Figure S1), confirming adequate covariate balance and supporting the validity of the matched estimate.

**TABLE 7:** Subgroup analysis: Sarcopenia and diabetic cardiomyopathy risk.

Subgroup	n	DCM: Sarcopenia n/N (%)	DCM: No Sarcopenia n/N (%)	Adjusted OR	95% CI	p value	p interaction
Overall	400	61/137 (44.5)	52/263 (19.8)	2.70	1.72–4.24	<0.001	—
Age (years)							
<65	204	19/72 (26.4)	15/132 (11.4)	2.70	1.54–4.73	0.001	0.258
≥65	196	42/65 (64.6)	37/131 (28.2)	3.80	2.18–6.62	<0.001	​
Sex							
Male	220	31/66 (47.0)	29/154 (18.8)	3.02	1.78–5.12	<0.001	0.644
Female	180	30/71 (42.3)	23/109 (21.1)	3.36	1.91–5.90	<0.001	​
HbA1c (%)							
<8.0	206	23/76 (30.3)	22/130 (16.9)	2.50	1.39–4.48	0.002	0.196
≥8.0	194	38/61 (62.3)	30/133 (22.6)	4.08	2.38–6.99	<0.001	​
DM duration (years)							
<10	232	23/80 (28.8)	26/152 (17.1)	2.84	1.58–5.10	<0.001	0.280
≥10	168	38/57 (66.7)	26/111 (23.4)	3.98	2.22–7.14	<0.001	​
eGFR (mL/min/1.73 m^2^)							
≥60	292	34/94 (36.2)	34/198 (17.2)	2.93	1.76–4.88	<0.001	0.330
30–59	108	27/43 (62.8)	18/65 (27.7)	3.92	2.04–7.52	<0.001	​

Adjusted ORs, from Model 3 (see Table 6), excluding the stratification variable. P interaction from likelihood ratio test. DCM, diabetic cardiomyopathy; DM, diabetes mellitus; eGFR, estimated glomerular filtration rate.

### 3.5 Inflammatory and molecular biomarkers

Sarcopenic patients showed significantly elevated inflammatory biomarkers (hs-CRP, ferritin, uric acid), cardiac stress markers (NT-proBNP, hs-Troponin I), and selected tumor markers (CEA, AFP, CA125), alongside reduced 25-OH vitamin D (Table 8, all p ≤ 0.006). HOMA-IR was also significantly higher in the sarcopenia group (5.5 vs. 3.9, p = 0.006), consistent with greater insulin resistance. Importantly, despite statistically significant between-group differences, the large majority of patients in both groups had tumor marker values within standard clinical reference ranges (CEA ≤ 5 ng/mL, AFP ≤ 20 ng/mL, CA125 ≤ 35 U/mL), indicating that these elevations likely reflect subclinical cytokine-driven upregulation (e.g., via IL-6 and TNF-α) independent of malignancy; active malignancy was rigorously excluded per pre-specified exclusion criterion 7. These biomarker differences suggest a systemic inflammatory and metabolic imbalance potentially linking skeletal muscle wasting to cardiac dysfunction.

**TABLE 8:** Inflammatory and molecular biomarkers by sarcopenia status.

Biomarker	All patients (n = 400)	Sarcopenia (n = 137)	Non-sarcopenia (n = 263)	p value
Inflammatory markers				
hs-CRP (mg/L), median (IQR)	2.9 (1.2–6.6)	4.8 (2.2–10.2)	2.2 (0.9–5.0)	<0.001
Ferritin (μg/L), median (IQR)	142.6 (88.4–224.8)	198.4 (128.6–310.2)	116.8 (72.4–186.4)	<0.001
Uric acid (μmol/L), mean ± SD	348.6 ± 94.2	392.4 ± 108.6	322.8 ± 82.4	<0.001
Tumor markers				
CEA (ng/mL), median (IQR)	3.2 (1.8–6.4)	4.6 (2.6–8.8)	2.6 (1.4–5.2)	<0.001
AFP (ng/mL), median (IQR)	4.8 (2.4–9.6)	6.2 (3.4–12.4)	4.2 (2.0–7.8)	<0.001
CA125 (U/mL), median (IQR)	14.6 (8.2–28.4)	18.8 (11.2–36.6)	12.4 (6.8–22.8)	<0.001
Metabolic markers				
Ferritin/transferrin ratio, median (IQR)	0.82 (0.54–1.28)	1.06 (0.72–1.58)	0.70 (0.44–1.08)	<0.001
HOMA-IR, median (IQR)	4.3 (2.9–7.8)	5.5 (3.5–9.4)	3.9 (2.5–7.0)	0.006
25-OH vitamin D (nmol/L), mean ± SD	44.4 ± 18.8	37.8 ± 16.0	47.9 ± 19.2	<0.001
Cardiac biomarkers				
NT-proBNP (pg/mL), median (IQR)	89.6 (43.2–186.8)	145.2 (70.0–302.4)	69.4 (35.0–140.8)	<0.001
hs-Troponin I (pg/mL), median (IQR)	6.6 (4.4–13.2)	9.0 (5.8–16.8)	5.4 (3.7–10.6)	<0.001

hs-CRP, high-sensitivity C-reactive protein; CEA, carcinoembryonic antigen; AFP, alpha-fetoprotein; CA125, cancer antigen 125; HOMA-IR, homeostasis model assessment of insulin resistance; NT-proBNP, N-terminal pro-B-type natriuretic peptide; hs-Troponin I, high-sensitivity troponin I.

## 4 Discussion

In this retrospective cohort of 400 T2DM patients enrolled over a four-year period (February 2022–February 2026), sarcopenia defined by AWGS 2019 criteria (Chen et al., 2020)—and corroborated by the updated AWGS 2025 criteria (Chen et al., 2025)—was present in 34.3% and was independently associated with a 2.70-fold increased risk of DCM after comprehensive confounder adjustment. This association was robust across all prespecified subgroups, and sarcopenic patients exhibited a distinctive biomarker profile featuring elevated inflammatory markers (hs-CRP, ferritin, uric acid) and cardiac stress markers, consistent with a shared systemic inflammatory muscle-heart dysfunction axis.

The sarcopenia prevalence of 34.3% in our hospital-based T2DM cohort is consistent with reported rates of 10%–35% in similar populations (Chen et al., 2020; Alfaro-Alvarado et al., 2023; Shatila et al., 2024; Lu et al., 2023; Tan et al., 2022; Haase et al., 2022). Our DCM prevalence of 28.3% aligns with published echocardiographic studies (Seferovic and Paulus, 2015; Seferović et al., 2024). The markedly higher DCM prevalence among sarcopenic T2DM patients (44.5%) suggests that sarcopenia screening may identify a high-risk subgroup warranting priority echocardiographic evaluation and early intervention. Moreover, the possibility of reverse causality warrants explicit consideration: impaired cardiac function and reduced exercise tolerance secondary to DCM may itself contribute to or accelerate skeletal muscle wasting, potentially amplifying the observed association. The cross-sectional design of this study cannot resolve this directionality; prospective longitudinal studies with serial assessments of both sarcopenia and cardiac function are needed to establish temporal precedence.

The biological mechanisms linking sarcopenia to DCM in T2DM are multifaceted. Chronic low-grade inflammation—reflected by elevated hs-CRP and ferritin in sarcopenic patients—simultaneously drives skeletal muscle protein catabolism and cardiac fibroblast activation (Tan et al., 2020; Mittal et al., 2021; Guo et al., 2026). Elevated uric acid levels observed in sarcopenic patients may further promote oxidative stress and impair both muscle anabolism and cardiac metabolic efficiency (Jia and Sowers, 2020; Chen et al., 2023). The subclinical elevations of CEA, AFP, and CA125 observed in the sarcopenia group—while remaining within standard clinical reference ranges in the majority of patients—may reflect cytokine-driven upregulation (particularly via IL-6 and TNF-α) of acute-phase and inflammatory pathways known to modulate these markers independently of malignancy. Active malignancy was rigorously excluded per pre-specified exclusion criterion 7, and these elevations are therefore most plausibly attributed to the chronic inflammatory milieu of sarcopenia rather than occult neoplastic disease. The precise mechanistic pathway warrants dedicated investigation in future studies. These findings suggest overlapping genetic and epigenetic regulatory mechanisms (Tan et al., 2020; Mittal et al., 2021).

From a genetic and genomic perspective, common inflammatory pathways—reflected by elevated ferritin and uric acid in our sarcopenic patients—may share upstream genetic regulators linking musculoskeletal and cardiac vulnerability in T2DM (Schaap et al., 2006). While the present dataset did not include genomic or epigenomic data and cannot directly test genetic mechanisms, the observed biomarker patterns are consistent with existing literature suggesting that shared epigenetic regulatory pathways may contribute to the muscle–heart dysfunction axis in T2DM (Zhao et al., 2026). Future research directions include investigating: (i) whether ACTN3 R577X polymorphism influences both skeletal and cardiac muscle architecture; (ii) whether hypermethylation of myogenic regulatory genes in T2DM coordinately impairs skeletal and cardiac muscle regeneration; and (iii) whether histone deacetylase-mediated suppression of anabolic signaling compounds concurrent musculoskeletal and cardiac vulnerability. These represent hypotheses for prospective integrative genomic investigations rather than conclusions of the present study.

The consistent subgroup findings (all p-interaction > 0.05) and validation by propensity score-matched analysis (matched OR = 2.44) reinforce the independence and robustness of the sarcopenia-DCM association. The numerically stronger association in patients with HbA1c ≥ 8.0% (OR = 4.08) and diabetes duration ≥ 10 years (OR = 3.98) suggests that sustained hyperglycemia amplifies muscle-heart crosstalk, potentially through advanced glycation end-products, oxidative stress, and accelerated protein catabolism (Jia and Sowers, 2020; Tan et al., 2020).

Clinical implications are significant: DCM remains underdiagnosed due to absent or nonspecific early symptoms, and no validated screening strategy currently exists for identifying T2DM patients at elevated DCM risk. Our findings suggest that sarcopenia screening using AWGS 2019 criteria (Chen et al., 2020)—and the refined approach endorsed by the AWGS 2025 update (Chen et al., 2025), which additionally incorporates calf circumference as a rapid screening surrogate—may serve as a cost-effective first step to stratify T2DM patients for priority cardiac evaluation. Exercise-based interventions targeting sarcopenia, which are known to reduce systemic inflammation and improve cardiac metabolic efficiency, may offer pleiotropic cardioprotective benefits (Chen et al., 2023; Severinsen and Pedersen, 2020; Ca et al., 2020; Nishikawa et al., 2021). SGLT2 inhibitors, used by 23.0% of our cohort, may additionally attenuate sarcopenia-DCM risk through dual metabolic and cardioprotective mechanisms, though this warrants dedicated prospective investigation (ElSayed et al., 2023; Shatila et al., 2024).

Several limitations of the present study warrant explicit acknowledgment. First, the retrospective single-center design at a Chinese tertiary hospital precludes causal inference and limits generalizability, as the cohort likely over-represents patients with more severe comorbidities, introducing potential selection bias; an external validation cohort would strengthen confidence in the findings. Second, sarcopenia was assessed at a single timepoint concurrent with DCM evaluation, precluding determination of temporal precedence; accordingly, the observed relationship should be interpreted as a cross-sectional association rather than a predictive relationship in the strict longitudinal sense. Third, and critically, DCM was diagnosed by Doppler echocardiography alone without cardiac MRI (CMR) confirmation: as acknowledged by the 2024 ESC DCM position statement, echocardiographic indices including GLS and diastolic parameters are susceptible to loading conditions and inter-operator variability, potentially affecting DCM classification accuracy; future studies should incorporate CMR for tissue characterization and more precise functional assessment. Fourth, while comparison of key baseline characteristics between the 49 excluded (incomplete data) and 400 included patients revealed no statistically significant differences, the assumption of missing-at-random inherent to complete-case analysis cannot be formally verified. Fifth, residual confounding from unmeasured variables applies, notably physical activity level (exercise intensity), dietary protein intake and nutritional status, and severity of diabetic complications (e.g., peripheral neuropathy)—all of which are closely associated with both sarcopenia and cardiac function—could not be captured from electronic medical records and therefore could not be adjusted for in the regression models. Sixth, generalizability may be constrained by the Chinese tertiary hospital setting. Prospective multicenter studies incorporating CMR-based DCM ascertainment, serial sarcopenia assessments, genetic profiling, and interventional arms are needed to confirm directionality and establish causality.

## 5 Conclusion

Sarcopenia, defined by AWGS 2019 criteria and corroborated by the updated AWGS 2025 framework, was independently associated with a 2.70-fold increased risk of diabetic cardiomyopathy in T2DM patients—representing a robust cross-sectional association, consistent across all prespecified subgroups and confirmed in propensity score-matched analysis; causal directionality requires prospective longitudinal confirmation. The distinctive biomarker profile of sarcopenic T2DM patients—featuring elevated inflammatory markers (hs-CRP, ferritin, uric acid) and cardiac stress markers alongside reduced vitamin D—supports a shared systemic inflammatory and metabolic muscle–heart dysfunction axis. The genetic and epigenetic mechanisms proposed to underlie this axis—including ACTN3 polymorphisms, myogenic gene hypermethylation, and HDAC-mediated signaling suppression—remain hypotheses to be tested in future integrative genomic studies (Zhao et al., 2026). Integrating AWGS 2019/2025 sarcopenia screening into routine T2DM management may facilitate earlier identification of patients at elevated DCM risk and inform targeted preventive strategies.

## Data Availability

The datasets generated and/or analyzed during the current study are available from the corresponding author on reasonable request.
