# Supplementary material for: Association between sarcopenia and the risk of diabetic cardiomyopathy in patients with type 2 diabetes mellitus: a single-center retrospective cohort study
Source: Front Genet. 2026 Aug 25;17:1892826. doi: 10.3389/fgene.2026.1892826 (PMC13549478; doi:10.3389/fgene.2026.1892826)
Supplement: Supplementary file 1 [file Supplementaryfile1.docx]

**Supplementary Material**

*Association between Sarcopenia and the Risk of Diabetic Cardiomyopathy in Patients with Type 2 Diabetes Mellitus: A Single-Center Retrospective Cohort Study*

Xia Liu, Lingjie Xu, Li Zhang, Hailin Yang, Qian Xiao*

**Supplementary Table S1**

| **Table S1. Covariate Balance Before and After Propensity Score Matching (1:2 Nearest-Neighbour, Caliper = 0.02)** | | | | | |
| --- | --- | --- | --- | --- | --- |
| *Matched variables: Age, Sex, HbA1c, Diabetes duration, eGFR, BMI \| Ratio 1:2 \| n(Sarcopenia) = 137, n(Non-sarcopenia) = 263 \| Balance threshold: SMD < 0.10* | | | | | |
| **Covariate** | **Sarcopenia** **(n = 137)** **Mean ± SD or %** | **Non-sarcopenia** **(n = 263)** **Mean ± SD or %** | **SMD** **Before** **Matching** | **SMD** **After** **Matching** | **Balance** **Achieved** **(SMD < 0.10)** |
| Age (years) | 69.1 ± 9.8 | 62.2 ± 9.9 | 0.700 | 0.042 | **Yes ✓** |
| Sex (male, %) | 48.2 | 58.6 | 0.210 | 0.031 | **Yes ✓** |
| HbA1c (%) | 8.4 ± 1.5 | 7.8 ± 1.3 | 0.427 | 0.058 | **Yes ✓** |
| Diabetes duration (years) | 11.4 ± 6.2 | 8.6 ± 5.5 | 0.478 | 0.047 | **Yes ✓** |
| eGFR (mL/min/1.73 m²) | 67.8 ± 19.4 | 74.9 ± 17.8 | 0.381 | 0.063 | **Yes ✓** |
| BMI (kg/m²) | 23.0 ± 3.3 | 25.6 ± 3.5 | 0.764 | 0.071 | **Yes ✓** |
| *Abbreviations: SMD, standardised mean difference; eGFR, estimated glomerular filtration rate; BMI, body mass index. SMD was calculated as Cohen's d for continuous variables and as (p₁ − p₂) / √[(p₁(1−p₁) + p₂(1−p₂))/2] for binary variables. All six matched covariates achieved post-matching SMD < 0.10, confirming adequate covariate balance.* | | | | | |

**Supplementary Figure S1**

*Love Plot: Standardized Mean Differences (SMDs) Before and After Propensity Score Matching*

*Figure S1. Love plot showing standardized mean differences (SMDs) for all six matched covariates before matching (red circles) and after 1:2 nearest-neighbour propensity score matching with a caliper of 0.02 (blue diamonds). The red dashed vertical line indicates the pre-specified balance threshold of SMD = 0.10. All six covariates achieved post-matching SMD < 0.10, confirming adequate covariate balance.*

*Abbreviations: eGFR, estimated glomerular filtration rate; BMI, body mass index; HbA1c, glycated haemoglobin; SMD, standardised mean difference.*
